# Fermented Foods and the Gut–Liver Axis: Modulation of MASLD Through Gut Microbiota

**DOI:** 10.3390/nu18030542

**Published:** 2026-02-06

**Authors:** Agnieszka Wesołek-Leszczyńska, Dawid Rosiejka, Kalina Bogdańska, Paweł Bogdański

**Affiliations:** 1 Department of Obesity Treatment, Metabolic Disorders, and Clinical Dietetics, Poznan University of Medical Sciences, 60-569 Poznań, Poland; 2 Doctoral School, Poznan University of Medical Sciences, 60-569 Poznań, Poland; 3 Medical Education, Wroclaw Medical University, 50-367 Wroclaw, Poland

**Keywords:** fermented foods, gut microbiota, gut-liver axis, metabolic dysfunction-associated steatotic liver disease, MASLD, short-chain fatty acids, intestinal barrier, bile acids, probiotics, prebiotics, personalized nutrition

## Abstract

**Background/Objectives**: Metabolic Dysfunction-Associated Steatotic Liver Disease (MASLD) is a prevalent condition defined by hepatic fat accumulation, inflammation, and metabolic dysregulation. Current evidence demonstrates that gut microbiota and their metabolites are associated with MASLD pathogenesis. Fermented foods, rich in live microbes and bioactive compounds, actively modulate the gut–liver axis and influence disease progression. This narrative review provides a comprehensive summary of current evidence on the impact of fermented foods on gut microbiota, intestinal barrier function, and gut–liver interactions, and demonstrates their potential role in preventing or mitigating MASLD. **Methods**: A comprehensive literature search of preclinical and clinical studies was conducted. Specifically, the review focused on fermented-food interventions, modulation of gut microbiota, metabolite production, and effects on hepatic metabolism and inflammation. **Results**: This review found that fermented foods provide probiotics, prebiotics, short-chain fatty acid (SCFAs), and bioactive compounds that enhance microbial diversity, improve intestinal barrier integrity, reduce endotoxemia, and modulate bile acid and lipid metabolism. Evidence from animal and human studies indicates that fermented food consumption can attenuate hepatic steatosis, inflammation, and metabolic dysregulation, with variability depending on individual microbiome composition. **Conclusions**: Altogether, these findings suggest that fermented foods represent a promising adjunctive dietary strategy for MASLD by modulating the gut–liver axis and supporting metabolic and hepatic health. Personalized approaches and further long-term clinical trials are required to optimize interventions and establish evidence-based recommendations.

## 1. Introduction

### 1.1. Fermented Foods

Fermented foods are defined as “foods or beverages produced through controlled microbial growth, and the conversion of food components through enzymatic action” [1]. Many products undergo fermentation. These include meat, fish, dairy products, vegetables, fruits, cereals, soybeans, and other legumes. Historically, food fermentation was used as a preservation method. The production of antimicrobial metabolites, such as organic acids, ethanol, and bacteriocins, reduces the risk of contamination with pathogenic microorganisms. During fermentation, numerous biochemical and physical modifications occur in food components. These changes improve food safety and shelf life. Currently, fermentation is primarily used to improve organoleptic properties, such as flavor and texture [2].

The fermentation process is influenced by many variables, including the microorganisms involved, nutrients, and environmental conditions. These factors contribute to the diversity of fermented foods [3]. There are two fermentation methods. The first is the natural method, where microorganisms occur naturally in raw foods or processing environments. Examples include sauerkraut, kimchi, and some fermented soy products. The second method adds starter cultures to food. Examples are kefir, yogurt, cheese, and kombucha [2]. Various strains of bacteria participate in the fermentation process. Table 1 presents the most commonly used food fermentation methods and the bacterial strains used [1,3].

During food fermentation, in addition to changes in taste and aroma, many health-promoting substances are produced. Fermented foods provide probiotics, prebiotics, lactic acid bacteria (LAB), yeast, organic acids, ethanol, and antimicrobial compounds that help balance the gut microbiome and support digestion [4]. Fermentation also produces bioactive peptides and biogenic amines, converts phenolic compounds into active forms, and lowers antinutritional substances [3,5].

Fermented foods may support health by enhancing gut microbiome diversity and reducing inflammation. Not all microbes in fermented foods act as probiotics; they do so only when viable and present at sufficient levels. Their probiotics mainly include bacteria such as *Lactobacillus*, *Bifidobacterium*, and *Streptococcus*, as well as yeasts such as *Saccharomyces cerevisiae* [6]. Prebiotics are non-digestible ingredients that promote the growth of beneficial gut microbes, especially *Bifidobacteria* and *Lactobacilli.* They mostly consist of non-starch polysaccharides and oligosaccharides, which human enzymes poorly digest. Gut microbiota ferment these substrates to produce key metabolites—mainly short-chain fatty acid (SCFAs)—that influence lipid and glucose metabolism [7].

Consuming fermented foods may affect the gut microbiome [1,3].

### 1.2. Gut Microbiome

The gut microbiome is a diverse community of microorganisms in the gastrointestinal tract that plays a key role in the host’s health [6]. Disruptions in the gut microbiome can cause immune or digestive disorders, leading to diseases such as obesity, diabetes, allergies, and inflammatory bowel disease [8]. Beyond these conditions, dysbiosis, defined as the alteration or imbalance in microbial communities’ structure, composition, and function [9], may also cause major metabolic changes, including reduced SCFAs production, greater intestinal permeability, endotoxemia from lipopolysaccharides (LPS), impaired bile acid metabolism, altered tryptophan–kynurenine pathway activity, and disruptions in choline and branched-chain amino acid (BCAA) metabolism—changes that together affect inflammation, insulin sensitivity, and energy balance. Diet is one of the most important modifiable factors affecting the gut microbiome throughout life [1,6]. Increasingly, evidence indicates a link between the gut microbiome and Metabolic Dysfunction-Associated Steatotic Liver Disease (MASLD) [10].

### 1.3. MASLD

Metabolic Dysfunction-Associated Steatotic Liver Disease (MASLD) is a chronic liver condition. It is characterized by excessive fat accumulation in hepatocytes, not caused by significant alcohol consumption or other secondary liver diseases. MASLD is closely linked to metabolic dysfunction, including obesity, type 2 diabetes, insulin resistance, and dyslipidemia. The term MASLD has been proposed to highlight the metabolic roots of the disease. This distinguishes it from the older term non-alcoholic fatty liver disease (NAFLD) [11].

In light of this evolving understanding, MASLD has emerged as a major global health challenge, affecting approximately 25–30% of the adult population worldwide. Its prevalence exhibits marked geographic variability, with the highest burden observed in the Middle East and South America, where rates exceed 30%. This increasing prevalence is largely driven by the global rise in obesity, sedentary lifestyle, and metabolic comorbidities [10].

Accurate diagnosis of MASLD is essential and requires evidence of hepatic steatosis in combination with at least one metabolic risk factor. The assessment of liver fat and fibrosis involves a range of approaches. Non-invasive imaging techniques are commonly employed, with ultrasound representing the most widely used method, while magnetic resonance imaging (MRI) and controlled attenuation parameter (CAP) measurement via transient elastography provide more precise quantification of hepatic fat content. Blood tests, including liver enzymes, lipid profile, glucose, and insulin levels, support the metabolic assessment. Additionally, validated fibrosis scoring systems, such as FIB-4 and NAFLD fibrosis score, are utilized to estimate the risk of advanced fibrosis. Liver biopsy is reserved for cases with diagnostic uncertainty or when advanced fibrosis is suspected [12].

Treatment relies on lifestyle changes and fixing metabolic problems. Weight loss from diet and exercise lowers liver fat and inflammation. Although lifestyle modification remains the cornerstone of MASLD management, current evidence indicates that therapeutic strategies should not be limited to dietary interventions and physical activity alone. In parallel, optimal treatment of metabolic comorbidities is essential and should include pharmacological interventions when indicated. Incretin-based therapies, such as glucagon-like peptide-1 receptor agonists and dual incretin agonists (e.g., semaglutide, tirzepatide), have demonstrated beneficial effects on body weight, insulin resistance, and hepatic steatosis, particularly in individuals with type 2 diabetes and obesity. Additionally, bariatric surgery represents an effective therapeutic option for selected patients with MASLD and severe obesity, leading to substantial and sustained weight reduction and improvement of liver histology. Moreover, if locally approved and according to label indications, adults with non-cirrhotic metabolic-associated steatohepatitis (MASH) and significant liver fibrosis (stage ≥ 2) should be considered for MASH-targeted pharmacotherapy with resmetirom, which has demonstrated histological improvements in steatohepatitis and fibrosis with an acceptable safety and tolerability profile in phase 3 clinical trials.

Untreated MASLD can progress through a continuum of hepatic injury with significant clinical consequences. Initially, simple steatosis may persist asymptomatically; however, a subset of patients develops MASH, characterized by hepatocellular inflammation. Persistent inflammatory activity promotes fibrogenesis, ultimately leading to hepatic fibrosis and, in advanced stages, cirrhosis. Cirrhosis is associated with an increased risk of hepatic decompensation, portal hypertension, and hepatocellular carcinoma (HCC) [12].

Untreated MASLD is closely linked to systemic metabolic dysfunction, not just liver-specific outcomes. Individuals face a higher risk of developing cardiovascular disease, such as heart attack and stroke; chronic kidney disease, often leading to progressive loss of kidney function; and type 2 diabetes, characterized by persistent high blood sugar. These three diseases—cardiovascular disease, chronic kidney disease, and type 2 diabetes—are the leading causes of morbidity and mortality in this population. Accurate diagnosis is essential for risk stratification and management. Early intervention is key to preventing both liver-related and non-liver-related complications [11].

### 1.4. Aim

The aim of this narrative review is to summarize and critically evaluate current evidence on the role of fermented foods in modulating the gut microbiota and the gut–liver axis in the context of MASLD. Specifically, this review focuses on the mechanisms through which fermented foods and fermentation-derived metabolites influence intestinal barrier integrity, microbial composition and function, bile acid metabolism, immune signaling, and hepatic lipid and glucose homeostasis. By integrating findings from preclinical and human studies, this article highlights the potential of fermented foods as dietary strategies to modulate metabolic and inflammatory pathways involved in MASLD progression, while also identifying current limitations and gaps warranting further investigation. While several recent reviews have discussed the gut–liver axis in MASLD, the present review uniquely focuses on the mechanistic links between the consumption of fermented foods, gut microbiota modulation, and progression of metabolic-associated fatty liver disease [13,14,15].

### 1.5. Literature Search Methodology

To gather the relevant literature for this review, we conducted a structured search in four major scientific databases: PubMed, Scopus, ScienceDirect, and Google Scholar. The search focused on peer-reviewed articles published in English, prioritizing recent studies that contribute to the current understanding the influence of fermented foods and gut microbiota on MASLD modulation.

We used a combination of keywords and Boolean operators to refine the search results. The primary keywords included: “MASLD”, “MAFLD”, “NAFLD”, “MASH”, “NASH”, “metabolic dysfunction–associated steatotic liver disease”, “metabolic dysfunction–associated fatty liver disease”, “non-alcoholic fatty liver disease”, “non-alcoholic steatohepatitis”, “fermented foods”, “food fermentation”, “probiotic foods”, “functional foods”, “yogurt”, “kefir”, “kimchi”, “sauerkraut”, “kombucha”, “miso”, “tempeh”, “gut microbiota”, “gut microbiome”, “intestinal microbiota”, “intestinal microbiome”, “dysbiosis”, “gut–liver axis”, “microbiota–liver axis”, “microbiome–liver axis”, along with variations and related terms as necessary. Publications issued between January 2000 and December 2025 were considered for inclusion. Articles were selected based on their relevance to the research focus, prioritizing studies that provided empirical data, theoretical insights, or significant contributions to the field. While this is not a systematic review, our approach aims to provide a comprehensive and representative overview of the existing literature.

## 2. Impact of Fermented Foods on Gut Microbiota

Live and/or inactivated microorganisms present in fermented foods, as well as nutrients released during fermentation, may modulate the gut microbiome through multiple biochemical and metabolic mechanisms [6]. These include the production of organic acids (e.g., lactic, acetic, and propionic acids), which lower intestinal pH and selectively inhibit pathogenic bacteria while growing beneficial taxa such as *Lactobacilli* and *Bifidobacteria*. Fermented foods also provide microbial cell wall components (peptidoglycans, lipoteichoic acids, β-glucans) that can interact with pattern recognition receptors (e.g., Toll-Like Receptors/Nucleotide-binding Oligomerization Domain receptors—TLR2/NOD2), influencing mucosal immunity and promoting the maturation of epithelial barrier function [1]. Fermentation releases bioactive peptides with antimicrobial or immunomodulatory properties, increases the bioavailability of vitamins (particularly B-group vitamins and vitamin K2), and generates SCFAs and other metabolites that affect host physiology. SCFAs—especially acetate, propionate, and butyrate—serve as energy substrates for colonocytes, regulate histone acetylation through Histone Deacetylases (HDAC) inhibition, modulate inflammatory signaling via G-protein coupled receptors (GPCRs) (e.g., G-protein coupled receptor 41 GPR41, G-protein coupled receptor 43 GPR43, G-protein coupled receptor 109A GPR109A), and influence lipid, glucose, and bile acid metabolism. Fermentation-derived exopolysaccharides and oligosaccharides act as prebiotic substrates, enhancing microbial diversity and supporting cross-feeding interactions within the gut ecosystem. Collectively, these biochemical processes help maintain microbial balance, strengthen intestinal barrier integrity, and shift metabolic activity toward pathways associated with reduced inflammation and improved metabolic health [16].

**Figure 1:**
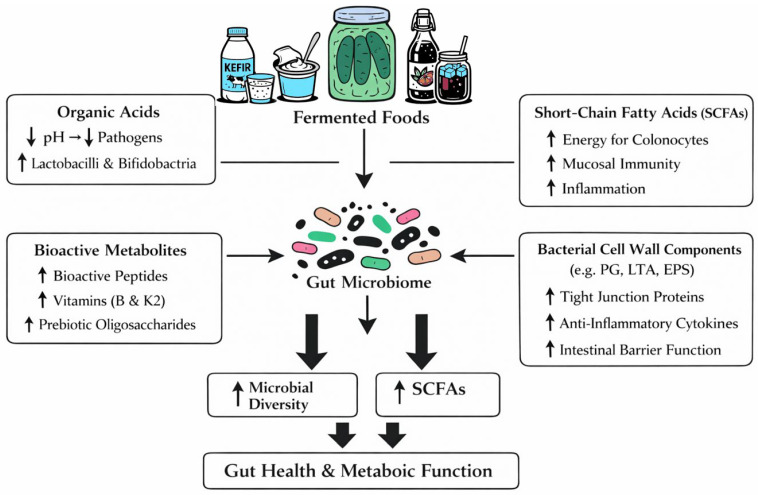
Impact of fermented cucumbers on gut microbiota and host metabolism. The diagram illustrates how pickled cucumbers (fermented foods) influence gut microbiota and host metabolic functions. Arrows indicate the effects of organic acids, SCFAs, bioactive metabolites, and bacterial cell wall components on gut microbial composition, SCFA production, intestinal barrier integrity, immune modulation, and metabolic pathways. Created by authors [1,2,3,5,7,16].

After consumption of fermented food, these microorganisms interact with the host microbiota, thereby influencing its health by modulating its composition and increasing microbial diversity and activity. To support gut health and overall metabolic function, include a variety of fermented foods in the diet on a regular basis [16]. In addition to changes in community composition, fermented foods appear to support intestinal barrier function and mucosal immunity. Preclinical and human studies report enhanced expression of tight junction proteins (e.g., occludin, claudin), reduced intestinal permeability, and decreased levels of pro-inflammatory cytokines such as Tumor Necrosis Factor alpha (TNF-α) and interleukin-6 (IL-6) [17,18]. An important element of this process is the bioconversion of key food components such as polysaccharides, proteins, lipids, and other dietary compounds into several metabolites with potentially beneficial health effects. This process produces SCFAs, bioactive peptides, exopolysaccharides, and vitamins [19]. This highlights the therapeutic potential of fermented foods in modulating the gut environment, increasing nutrient bioavailability, and promoting systemic health benefits. SCFAs produced as bacterial metabolites modulate gut immune function by interacting with immune cells, such as dendritic cells and T lymphocytes, and by increasing the production of anti-inflammatory cytokines. Furthermore, SCFAs, such as butyrate, may serve as a primary energy source for colonic epithelial cells, thereby increasing barrier integrity and reducing intestinal permeability [16,20]. The impact of fermented foods on gut microbiota is shown in Figure 1.

## 3. Fermented Foods and the Gut–Liver Axis

The gut–liver axis is a dynamic communication pathway that connects the intestinal microbiota, the gut barrier, and the liver. Fermented foods can modulate this axis in several ways. The gut–liver axis is often seen as a unidirectional pathway from the intestines to the liver. However, the liver also communicates directly with the intestines through the biliary system. Both intestinal and liver diseases are often linked to dysregulated gut–liver communication and dysbiosis [21]. This condition, together with a disrupted intestinal barrier, impaired immune homeostasis, and bile salt imbalance, may influence increased intestinal permeability and systemic inflammation [22]. The gut microbiota plays an important etiopathogenic role in intestinal and liver diseases. These diseases also negatively impact the gut microbiota [23]. The significance of the gut–liver axis goes beyond the microbiome and bacterial spread. An additional communication pathway, the systemic circulation, allows gut–liver mediators such as cytokines, hormones, and bile salts to directly link the organs’ functions. In this way, the intestines and liver interact, which is important for both organs to function [13].

### 3.1. Gut Microbiota Metabolites

Fermented foods introduce live microbes and generate metabolites, including SCFAs, organic acids, and modified polyphenols. Importantly, these metabolites reach the liver via the portal circulation and influence metabolism by activating various receptors (e.g., GPR41/43, Farnesoid X Receptor—FXR/Takeda G-protein Receptor 5—TGR5) and signaling pathways (e.g., AMP-activated protein kinase—AMPK, peroxisome Proliferator-Activated Receptors—PPARs), thereby reducing lipogenesis and improving fatty-acid oxidation [23,24]. In addition to these metabolic effects, fermented foods also may support gut barrier integrity and reduce translocation of microbial-derived inflammatory signals (LPS, peptidoglycan) into the portal vein. Consequently, this reduction in endotoxin load may lower hepatic Kupffer cell activation and the release of pro-inflammatory cytokines (e.g., TNF-α, IL-6) [18,25]. Finally, fermentation-derived bioactive compounds exert antioxidant and anti-inflammatory effects in the liver, reducing oxidative stress and protecting hepatocytes from damage [21,24].

### 3.2. Bile’s Role

Liver cells produce and secrete bile, a substance rich in Immunoglobulin A (IgA), bicarbonates, antimicrobial molecules, and bile acids. Bile acids serve numerous functions, such as facilitating the digestion and absorption of lipids and lipid-soluble vitamins and modulating the composition of the gut microbiota. They exert bacteriostatic effects both directly through their detergent properties and indirectly by activating the bile acid receptor, the farnesoid X receptor (FXR). In this way, they enhance the production of antimicrobial molecules in intestinal epithelial cells and maintain intestinal barrier integrity [23,26].

**Figure 2:**
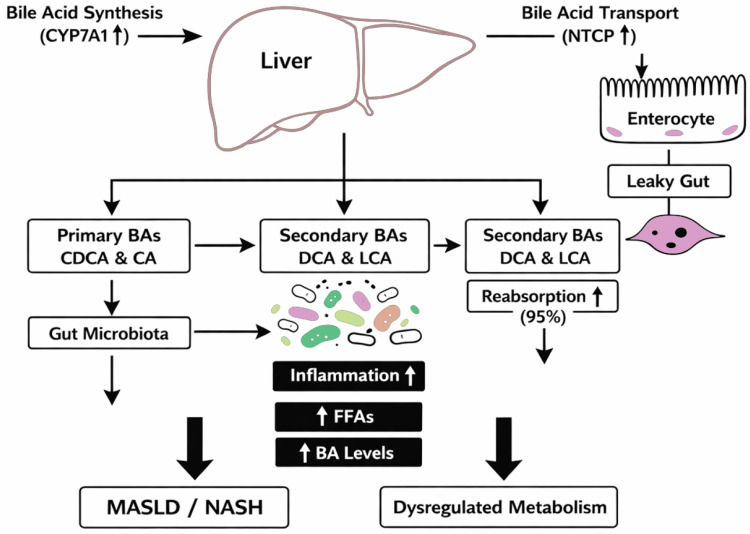
Bile acid metabolism and its role in MASLD/NASH progression. Abbreviations: BAs—Bile Acids; CDCA—Chenodeoxycholic Acid; CA—Cholic Acid; DCA—Deoxycholic Acid; LCA—Lithocholic Acid; FFAs—Free Fatty Acids; MASLD—Metabolic dysfunction-Associated Steatotic Liver Disease; NASH—Non-Alcoholic Steatohepatitis; SHP—Small Heterodimer Partner; CYP7A1—Cholesterol 7 Alpha-Hydroxylase; NTCP—Sodium Taurocholate Co-transporting Polypeptide. The diagram illustrates the synthesis of primary bile acids (CDCA, CA) in the liver, their conversion into secondary bile acids (DCA, LCA) by the gut microbiota, and re-absorption in the ileum. Dysbiosis and impaired bile acid transport disrupt this balance, leading to leaky gut, increased free fatty acids (FFAs), inflammation, and dysregulated metabolism, which contribute to the development and progression of metabolic dysfunction-associated steatotic liver disease (MASLD) and non-alcoholic steatohepatitis (NASH). Created by authors [13,21,22,23,24,25,26,27,28,29,30,31].

Studies with rats have shown that interruption of bile flow into the intestine by bile duct ligation or cholecystoductal fistula was associated with an increase in the population of aerobic Gram-negative bacteria in the cecum and increased bacterial translocation [27]. In human studies, inhibition of endogenous bile acid synthesis by obeticholic acid, a bile acid analogue and FXR agonist, led to the proliferation of Gram-positive bacteria of the small intestine [28]. However, the microbiota can also modulate bile acid supply and composition by deconjugating and dehydroxylating primary liver-derived bile acids into secondary bile acids, which are less efficiently reabsorbed in the intestine [23,29]. Stimulation in the ileum by bile acids of the FXR target gene, fibroblast growth factor (FGF) 15 in mice and FGF19 in humans, which, in addition to inhibiting hepatic bile acid synthesis in a self-regulatory feedback loop, affects metabolic functions such as regulation of insulin sensitivity and stimulation of hepatic protein and glycogen synthesis [30,31]. The synthesis of primary bile acids in the liver, their conversion into secondary bile acids by the gut microbiota, and reabsorption in the ileum is shown in Figure 2.

## 4. Gut–Liver Axis in MASLD Pathogenesis

### 4.1. Gut Barrier Integrity

Gut integrity is essential for maintaining homeostasis within the organism’s internal environment [32]. In modern research, the intestinal epithelial barrier is recognized for facilitating efficient nutrient absorption and for limiting the passage of potentially harmful microorganisms and their products from the gut lumen into the circulation [22,32]. Compromise of this barrier may result from gut dysbiosis, which serves as both a causative and predisposing factor in hepatic diseases such as MASLD [22]. Current external factors, including widely used food preservatives such as methylparaben and ethylparaben, have been identified as significant contributors to this bacterial imbalance. Disruptions in the gut microbiota alter its composition and metabolic activity, thereby promoting the onset of a pathological condition known as “leaky gut.” This process is characterized by decreased expression of the tight junction protein Zonula Occludens-1 (ZO-1), leading to increased intestinal permeability [33].

### 4.2. Bile Acids

Bile acids (BAs) function as signaling molecules that regulate their own synthesis and transport while also modulating essential pathways involved in lipid and glucose regulation [29].

Their biosynthesis occurs in the liver, where they are classified into primary BAs (chenodeoxycholic acid [CDCA] and cholic acid [CA]) and secondary BAs (deoxycholic acid [DCA] and lithocholic acid [LCA]) [34]. Secondary BAs, which are more hydrophobic, are generated from primary bile acids through biotransformation by the gut microbiota [34,35]. In ileal enterocytes, around 95% of the secondary BAs are reabsorbed by active transport and then enter the portal vein to reach the liver [36]. Dysfunction in BA biosynthesis triggers an inflammatory response in the liver, leading to the development of MASLD and progression to MASH. MASLD is associated with elevated free fatty acids (FFAs), which suppress Small Heterodimer Partner (SHP) expression. This suppression overactivates Cholesterol 7-alpha-hydroxylase (CYP7A1) and Na^+^-taurocholate co-transporting polypeptide (NTCP), resulting in increased bile acid synthesis. Furthermore, alterations in bile acid metabolism contribute to elevated BA concentrations in both the bloodstream and feces [29]. Additionally, gut dysbiosis may disrupt the balance between primary and secondary BAs, leading to leaky gut and disturbances in homeostasis [33]. Ultimately, weakening of the intestinal barrier, along with impaired BA transporter function, disrupts BA circulation and dysregulates receptor-driven energy metabolism [34].

### 4.3. Interaction Between Hepatic Inflammation, Insulin Resistance, and Microbiota

The development of MASLD involves interconnected disturbances in gut microbiota composition, inflammatory pathways, and insulin signaling [37]. Activation of the last two cells triggers downstream inflammatory signaling, characterized by the release of TNF-α, IL-1, IL-18, and C-X-C motif chemokine ligand (CXCL) chemokines, as well as increased reactive oxygen species production. Moreover, Kupffer cells and hepatocytes are stimulated by Pathogen-Associated Molecular Patterns (PAMPs) and Damage-Associated Molecular Patterns (DAMPs), thereby activating inflammatory processes. The dissemination of this inflammation in the liver is associated with metabolic disturbances, including enhanced bacterial ethanol production, altered handling of short-chain fatty acids, and reduced choline availability, which together contribute to hepatic fat accumulation, persistent inflammation, and fibrosis characteristic of MASLD [22,37].

Another consequence of the inflammatory process is insulin resistance (IR), which develops through the overproduction of pro-inflammatory cytokines. These cytokines, such as TNF-α, IL-1β, IL-6, and IL-8, are released following the interaction of LPS with TLR4 and CD14 within the liver. Progressive IR forms a self-perpetuating cycle with chronic inflammation. Heightened activity of pro-inflammatory cells, including M1-polarized Kupffer cells and M1 macrophages, enhances the release of Interleukin-1 beta (IL-1β) and other mediators, which, in turn, stimulate hepatic triglyceride production and further aggravate IR [38]. At the same time, adipose tissue insulin resistance accelerates lipolysis, increasing the influx of free fatty acids (FFAs) into the liver [39].

Overaccumulation of FFAs in hepatocytes, overactivated de novo lipogenesis, and the overload of disposal pathways—mitochondrial β-oxidation and the export of triglycerides as very-low-density lipoproteins (VLDL)—are mechanisms that can become saturated and cause triglycerides to accumulate in the liver, leading to hepatic steatosis [35].

FFA β-oxidation is tightly linked to the development of hepatic immune-mediated inflammation, as increased metabolic activity generates reactive oxygen species (ROS). The increase in oxidative stress activates the immune mechanism within hepatocytes, driving cellular injury and death [39]. Thus, MASLD emerges as a multifactorial disorder arising from the convergence of gut–liver axis dysfunction, chronic inflammation, and metabolic derangements [37].

### 4.4. Role of Dysbiosis and Microbial Metabolites

MASLD progression is closely linked to the composition of the gut microbiota and the biological activity of its metabolites. Increased bacteria, such as *Enterobacteriaceae*, *Escherichia coli*, and *Shigella*, together with changes in the production of short-chain fatty acids, lipopolysaccharide, trimethylamine-N-oxide, and alcohol, lead to the worsening of disease [34,40].

Carbohydrates that reach the intestine as undigested dietary residues undergo fermentation by the gut microbiota, SCFAs, which regulate metabolic homeostasis by stimulating the release of peptides such as Glucagon-Like Peptide 1 (GLP-1), Peptide YY (PYY), Fasting-Induced Adipose Factor (FIAF), and Yin Yang 1 (YY1). These effects are responsible for intestinal barrier integrity and enhance insulin secretion and sensitivity. Moreover, butyrate may influence gut homeostasis by fueling β-oxidation, supporting the anaerobic environment required for balanced microbial communities, and activating Peroxisome Proliferator-Activated Receptor gamma (PPARγ); it also suppresses nitric oxide (NO) synthase, lowering NO levels and limiting the expansion of Enterobacteriaceae. Additionally, butyrate promotes anti-inflammatory responses by activating immune cells [40]. Bacteria such as *Faecalibacterium*, *Akkermansia*, *Roseburia*, and *Bacteroides* produce SCFAs in the gut [41]. A reduction in these microbial populations may decrease SCFAs levels, which may influence MASLD progression by impairing GLP-1 secretion, increasing intestinal permeability, and exacerbating endotoxemia [35].

Weakening of the intestinal barrier also allows lipopolysaccharide (LPS) to translocate to the liver via the portal vein [37]. LPS, the main bioactive component of endotoxins produced by Gram-negative bacteria, reaches the liver and activates TLR4 and CD14 receptors. This activation triggers downstream inflammatory signaling, which plays a crucial role in the progression of MASLD [34,38].

Choline and carnitine, found for example in eggs, are converted by the gut microbiota into trimethylamine (TMA), which is then transported to the liver [42]. There, TMA is oxidized to trimethylamine N-oxide (TMAO) by the enzyme Flavin-containing Monooxygenase 3 (FMO3) [33]. Only about 1% of gut bacteria can metabolize choline and carnitine, making the elevated TMAO levels observed in MASLD patients highly dependent on gut microbiota composition. This progression is correlated with increased quantities of bacteria, such as Firmicutes and Proteobacteria, as observed. These groups include key microorganisms capable of producing TMA, thereby increasing hepatic TMAO production [42].

A clear association has been demonstrated between circulating TMAO levels and both MASLD severity and mortality [42]. Elevated concentrations of TMAO and its precursor, TMA, promote hepatic inflammatory responses, leading to liver steatosis and a poor prognosis in MASLD. Increased TMAO levels also promote the progression of liver fibrosis. Moreover, TMAO is responsible for atherosclerosis and systemic inflammation by altering genes involved in MASLD pathogenesis. A bacterial metabolite that also contributes to MASLD progression is ethanol, produced in the gut by fermentation by specific microorganisms [33]. The increase in alcohol-producing bacteria, such as certain *Escherichia species* within the *Proteobacteria phylum*, can be promoted by a high-fat diet [43].

Ethanol produced in the intestine is transported to the liver, where it promotes several pathological effects. It causes oxidative stress and hepatocyte injury, and enhances inflammatory signaling by increasing the production of pro-inflammatory cytokines. Furthermore, ethanol metabolites are associated with hepatic steatosis by activating lipogenesis through the modulation of lipid-related signaling pathways and specific gene expression [33]. Nutrients and Microbial Metabolites Involved in the Gut–Liver Axis and MASLD Pathogenesis summary are presented in Table 2.

## 5. MASLD and Food Fermentation—Studies

MASLD is a chronic condition characterized by excessive fat accumulation in liver cells. It is a progressive metabolic disorder that encompasses a spectrum of conditions, from ectopic triglyceride (TG) accumulation in the liver (simple steatosis) to steatohepatitis (steatohepatitis associated with metabolic dysfunction, MASH), liver fibrosis, and cirrhosis [40]. MASLD is one of the most common liver diseases, affecting approximately 32% of the population. Its incidence is increasing with the rise in obesity and type 2 diabetes. Approximately one-quarter of MASLD cases progress to MASH, increasing the risk of liver and cardiovascular morbidity and mortality [22]. The pathogenesis of MASLD is complex and multifactorial, involving both genetic and environmental factors [13]. Diet is now recognized to play a key role in the development of chronic liver disease (CLD) and is also a fundamental component of its treatment. Recent research suggests that a diet high in sugar, saturated fat, and cholesterol contributes to the development and onset of MASLD [44]. Fat accumulation, liver cell damage, and intestinal barrier dysfunction are key components of the pathophysiology of MASLD. A healthy gut plays a crucial role in the absorption of essential nutrients and in preventing the entry of microorganisms from the intestinal lumen. Intestinal barrier dysfunction increases intestinal permeability, which significantly contributes to the initiation and progression of intra- and extrahepatic damage in MASLD [26].

Studies have shown that increased intestinal mucosal inflammation and damage to the intestinal epithelial barrier increase the likelihood of microbial translocation into the portal circulation, thereby exacerbating inflammation by activating macrophages and Kupffer cells, which contribute to MASLD [22,45,46,47]. MASLD is associated with increased circulating LPS levels, leading to bacterial overgrowth in the small intestine, disruption of tight junctions, and impaired intestinal permeability, which, in turn, facilitates bacterial translocation [26]. In MASLD, gut dysbiosis is characterized by changes in the composition and diversity of the gut microbiome [48].

Most studies have shown that individuals with MASLD have an increased abundance of Gram-negative bacteria *Bacteroidetes*, resulting in a reduced *Firmicutes-to-Bacteroidetes* (F/B) ratio. Compared with healthy controls, MASLD patients have increased *Enterobacterales* and *Proteobacteria*, while decreased *Akkermansia muciniphila* (*A. muciniphila*) and *Faecalibacterium prausnitzii* (*F. prausnitzii*). This may be related to the decreased number of tight junctions (TJs) demonstrated in mice fed a high-fat diet (HFD). TJs protect the intestine against invasion by pathogen-associated molecular patterns (PAMPs). Animal models of MASLD have been shown to significantly reduce TJ numbers when fed an HFD. This reduction in TJ numbers may contribute to increased PAMP permeability and gut leakiness [13,49,50,51]. Importantly, the HFD increases the levels of some unfavorable bacteria, such as *Enterobacter cloacae B29*, *Escherichia coli py102*, and *Klebsiella pneumoniae A7*. These bacteria have been implicated in the progression of MASLD. In later stages of the disease, populations such as *Proteus* and *Escherichia coli* may increase, while *Firmicutes* are significantly reduced [40]. Changes in the composition and richness of the gut microbiome differ between patients with MASLD and those with obesity compared with those with normal weight [48]. Studies have shown that Asian patients with MASLD and obesity had distinct microbial signatures compared to Asian patients with normal weight and MASLD. Patients with MASLD and obesity had lower levels of *Ruminococcaceae* and increased abundance of *Veillonellaceae*, and also demonstrated low gut microbiome diversity. Furthermore, these changes were associated with the severity of fibrosis [52].

Trimethylamine N-oxide (TMAO), a choline metabolite produced by the gut microbiota, is also associated with the development of MASLD [53]. Specifically, TMAO contributes to hepatic fat accumulation by inhibiting bile acid-dependent hepatic Farnesoid X Receptor (FXR) signaling [54]. Furthermore, elevated serum TMAO levels have been associated with the severity of fatty liver disease and higher all-cause mortality in individuals with MASLD [55]. In addition to these effects, TMAO acts as an agonist of the PERK pathway, a branch of the ER stress pathways that promotes MASLD. Beyond its signaling roles, Trimethylamine N-oxide also contributes to the progression of MASLD by exacerbating gut barrier dysfunction. Recent evidence indicates that intestinal interleukin-33 increases gut microbiota-derived TMAO synthesis and exacerbates MASLD progression through dual regulation of hypoxia-inducible factor-1α (HIF-1α) [26]. Collectively, these findings underscore that TMAO has been proposed as a risk factor in the development of MASLD [13].

SCFAs, mainly acetate, propionate, and butyrate, are key microbial metabolites linking gut microbiota composition with liver metabolism in MASLD. Recent studies have shown that SCFAs exert multifaceted effects on hepatic lipid and glucose homeostasis through receptor-mediated and epigenetic mechanisms [56,57]. Intestinal dysbiosis may increase the severity of MASLD by reducing SCFAs. In the gut–liver axis, SCFAs reach the liver via the portal vein, where they activate G-protein coupled receptors (GPR41 and GPR43) and modulate energy metabolism through AMP-activated protein kinase (AMPK) signaling, resulting in decreased de novo lipogenesis and enhanced fatty acid oxidation. Butyrate, in particular, serves as an energy source for colonocytes and strengthens the intestinal barrier by upregulating tight junction proteins, thereby reducing endotoxin (LPS) translocation to the liver and subsequent Kupffer cell activation [57]. Propionate can influence hepatic gluconeogenesis, while acetate contributes to Acetyl coenzyme A (acetyl-CoA) pools and may also fuel lipogenesis, depending on the metabolic context [56].

Beyond their role in metabolic regulation, SCFAs have anti-inflammatory and immunomodulatory effects. By inhibiting histone deacetylases (HDACs), they promote regulatory T-cell differentiation and suppress the expression of pro-inflammatory cytokines such as TNF-α and IL-6, mitigating hepatic inflammation associated with MASLD [57,58]. Animal studies demonstrate that SCFA supplementation or microbiota-targeted interventions that increase SCFA production alleviate steatosis, oxidative stress, and markers of fibrosis in liver tissue [59]. Human metabolomic and observational studies further support this link, showing that altered circulating SCFA profiles correlate with disease severity and fibrosis stage in MASLD patients [57]. However, findings remain inconsistent across cohorts, possibly reflecting dietary variation, microbial diversity, and differing SCFA ratios. While increased intestinal SCFA production generally correlates with metabolic benefits, excessive acetate flux under insulin-resistant conditions may paradoxically contribute to hepatic lipogenesis [56,57].

A summary is presented in Table 3.

Current evidence suggests that maintaining a balanced SCFA profile-favoring butyrate- and propionate-producing bacteria-helps maintain gut barrier strength and metabolic resilience, thereby countering MASLD progression.

## 6. Modulation of MASLD Through Gut Microbiota

Recent evidence from preclinical and clinical studies indicates that fermented foods can enhance hepatic lipid metabolism, decrease inflammation, and positively influence gut microbial composition in MASLD. While these benefits are observed through actions along the gut–liver axis, their extent and reliability depend on the specific fermented food, its microbes, and the duration of the intervention. In animal models of diet-induced hepatic steatosis, supplementation with fermented dairy products, such as kefir, has been shown to attenuate lipid accumulation, improve markers of hepatic oxidative stress, and normalize levels of inflammatory cytokines. Studies have shown the positive effects of kombucha on the body, particularly by modulating the gut–liver–metabolic axis [60].

In mice with diet-induced obesity and MASLD, kombucha supplementation significantly improved glucose tolerance, ameliorated hyperinsulinemia, and ameliorated hepatic steatosis. This effect was associated with reduced expression of proinflammatory genes such as TNF-α and Sterol Regulatory Element-Binding Protein 1 (SREBP-1), reduced collagen deposition in liver tissue, and restored insulin signaling via Protein Kinase B (AKT) phosphorylation, suggesting potent anti-inflammatory and hepatoprotective effects [61].

In a Lee et al. study, methionine/choline-deficient MASH mice supplemented with kombucha showed significant reductions in hepatic triglyceride levels, inflammation, and fibrosis. Kombucha promoted liver cell survival by reducing apoptosis and increasing cell proliferation. It inhibited lipid accumulation by downregulating genes such as Cluster of Differentiation 36, Cd36; Peroxisome Proliferator-Activated Receptor gamma, Pparγ; Fatty Acid Synthase, Fas; and Sterol Regulatory Element-Binding Protein, 1c Srebp1c. It also stimulated β-oxidation by upregulating Peroxisome Proliferator-Activated Receptor Gamma Coactivator 1-alpha, Ppargc1α; Carnitine Palmitoyltransferase, 1 Cpt1; and Acyl-CoA Oxidase 1, Acox1. Importantly, kombucha interacts with bile acid receptors, including TGR5 and FXR, thus extending its beneficial effects on lipid regulation and immune modulation. These hepatoprotective effects highlight how kombucha modulates both metabolic and inflammatory pathways [62].

In a study by Hyun et al., administration of kombucha to mice fed a methionine/choline-deficient diet for 4 weeks ameliorated macromolecular fatty liver disease. Furthermore, triglyceride, Alanine Aminotransferase (ALT), and Aspartate Aminotransferase (AST) levels decreased, and Ribonucleic Acid (RNA) expression showed decreased triglyceride synthesis and fatty acid uptake. These results indicate that kombucha limits lipid accumulation and protects the liver from damage, promoting liver regeneration in mice [63]. Another study in rats fed a high-fat, high-fructose diet evaluated the effects of administering kombucha with green or black tea for 10 weeks. Kombucha improved glucose metabolism, plasma total antioxidant capacity, superoxide dismutase activity, and reduced nitric oxide concentration. Both types of kombucha reduced inflammation by lowering the neutrophil-to-lymphocyte ratio (NLR), reduced total body fat and blood triglyceride levels, reversed hepatic steatosis (from grade 2 to 1), and also modulated genes related to adipogenesis and β-oxidation [64].

In a 10-week randomized controlled trial in healthy adults, Wastyk et al. showed that daily consumption of fermented foods increased microbiome diversity and reduced levels of 19 inflammatory markers, including interleukin (IL)-6 and IL-12b, suggesting their immunomodulatory and anti-inflammatory properties [65].

Han et al. reported that consumption of fermented kimchi reduced metabolic markers and altered the gut microbiota in women with overweight [66], while another kefir-based intervention reduced inflammatory symptoms in patients with inflammatory bowel disease [67].

In a study on MASLD and metabolic syndrome, Chen et al. found that yogurt consumption significantly improved insulin sensitivity and reduced liver fat accumulation in women with obesity [68]. Consuming amazake, a traditional Japanese fermented rice beverage, has been shown to reduce serum TNF-α levels and alleviate symptoms such as muscle spasms and depression in patients with MASLD and periodontal disease, highlighting its potential as an anti-inflammatory dietary intervention that improves quality of life [69].

In a randomized controlled trial involving 80 adults with MASLD, daily kefir supplementation for eight weeks resulted in improved HDL cholesterol and reduced systemic inflammation, although serum transaminases remained unchanged compared to the control group [70].

Despite promising evidence, several limitations should be acknowledged. Most human studies have short intervention durations (≤12 weeks), small sample sizes, and considerable variation in fermented food matrices, microbial strains, and dosages, which limits comparability and reproducibility [18]. Moreover, baseline gut microbiota composition differs substantially among participants, thereby influencing individual responses to fermented foods. In some cases, excessive intake of carbohydrate-rich fermented products may contribute to energy surplus and lower potential metabolic benefits. Future clinical trials should therefore standardize fermented food preparations, integrate multi-omics approaches to characterize microbiota and metabolite shifts, and use imaging-based assessments to quantify hepatic fat changes objectively.

Current data indicate that fermented foods represent a promising, low-cost dietary strategy to modulate metabolic and inflammatory pathways in MASLD. Consistent consumption appears to enhance gut microbial diversity, increase SCFA production, and improve gut barrier integrity, thereby reducing hepatic fat accumulation and inflammation. However, definitive conclusions require larger, long-term randomized trials designed to isolate the effects of specific fermented food components within the context of a controlled dietary pattern.

A summary is presented in Table 4.

## 7. Practical and Clinical Implications

Fermented foods have gained increasing attention as dietary components with likely positive effects for gut and metabolic health; however, standardized dietary recommendations and harmless use thresholds remain insufficiently defined. Current national and international dietary guidelines generally recognize fermented foods as part of healthy dietary patterns, such as the Mediterranean and traditional Asian diets, but do not specify quantitative intake recommendations. This lack of consensus largely reflects the heterogeneity of fermented food products, differences in microbial composition, fermentation methods, and variability in bioactive compound content.

Evidence from observational studies and short-term intervention trials suggests that regular consumption of fermented foods-usually varying from one to two servings per day-is well tolerated in healthy individuals and may support gut microbiota diversity and immune regulation. Fermented dairy products, such as yogurt and kefir, are the most extensively studied and are generally considered safe when consumed in moderate amounts as part of a well-rounded diet. Non-dairy fermented foods, including fermented vegetables, soy-based products, and fermented beverages, are typically safe for most people, though their nutritional profiles may vary widely in terms of sodium, sugar, and alcohol content.

Safety considerations are notably pertinent to specific populations. Persons with reduced immune function, advanced liver disease, or increased intestinal permeability may be more susceptible to adverse effects from excessive intake of live microorganisms or biogenic amines. Additionally, some fermented foods—especially fermented vegetables and condiments—contain high levels of sodium, which may increase hypertension and cardiometabolic risk when consumed in large quantities. Fermented beverages such as kombucha may contain variable amounts of ethanol and organic acids, necessitating moderation, in particular for those with MASLD.

Fermented foods may complement pharmacological therapies for MASLD by modulating the gut–liver axis. Fermentation-derived metabolites, including SCFAs, bioactive peptides, and polyphenols, can enhance intestinal barrier integrity, reduce endotoxemia, and attenuate hepatic inflammation. They may augment the effects of drugs such as bile acid modulators (e.g., obeticholic acid) or insulin-sensitizing agents (e.g., metformin). Concurrent modulation of FXR/TGR5 signaling, SCFA-mediated metabolic improvements, and reduced oxidative stress may produce synergistic benefits. However, individual variability in microbiome composition and host factors can influence responses, and clinicians should exercise caution in immunocompromised patients or when patients consume high doses of live microbial products. Researchers should further investigate personalized strategies that integrate diet and pharmacotherapy.

From a clinical and public health perspective, fermented foods should be regarded as auxiliary factors of dietary patterns rather than as therapeutic agents. Emphasis should be placed on product quality, microbial viability, minimal processing, and the diversity of fermented food sources to reduce the risk of excessive exposure to any single bioactive compound. Subsequent research should aim to establish evidence-based intake ranges, clarify long-term safety profiles, and identify population-specific recommendations, particularly for individuals with metabolic and liver-related disorders.

## 8. Conclusions

Fermented foods modulate gut microbiota composition and function. They enhance intestinal barrier integrity and influence metabolites that affect the gut–liver axis. These microbiome-mediated effects may help prevent and reduce MASLD. Integrating fermented foods into dietary strategies, possibly in combination with pharmacological therapies, is a promising approach to support metabolic and liver health. Personalized approaches and more long-term studies are still needed.

## Figures and Tables

**Table 1:** Food fermentation methods and bacterial family involved [1,3].

Fermentation Type	Bacterial Family Involved	Main Products	Main Produced Fermented Foods
Lactic	*Lactobacillaceae*, *Leuconostocaceae*, *Streptococcaceae*	Lactic acid (homolactic), CO_2_, ethanol (heterolactic)	Dairy (yogurt, cheeses, kefir), sauerkraut, kimchi, pickles, tempeh, fermented meats
Alcoholic	*Saccharomyces* spp., *Kloeckera* spp.	Ethanol, CO_2_	Wine, beer, kefir
Acetic	*Acetobacter* spp., *Gluconacetobacter*, *Gluconobacter*	Acetate, EPS	Chocolate, coffee, vinegar, specialty beers, water kefir
Propionic	*Propionibacterium* spp.	Propionate, acetate, CO_2_, succinate (Wood-Werkman pathway)	Swiss-type cheeses

CO_2_—carbon dioxide; EPS—specific exopolysaccharide.

**Table 2:** Nutrients and Microbial Metabolites Involved in the Gut–Liver Axis and MASLD Pathogenesis [25,33,34,35,37,38,40,41,42,43].

Nutrient/Metabolite	Source/Production	Impact on Gut Barrier	Impact on Liver/MASLD	Mechanisms
SCFAs: butyrate, acetate, propionate	Fermentation of dietary fibers by gut bacteria (*Faecalibacterium*, *Akkermansia*, *Roseburia*, *Bacteroides*)	Strengthen barrier, maintain anaerobic environment, fuel β-oxidation	Anti-inflammatory, improve insulin sensitivity, reduce hepatic fat accumulation	Stimulate GLP-1, PYY, FIAF; activate PPARγ; suppress iNOS → ↓ NO; maintain gut–liver homeostasis
Choline	Eggs, meat, fish; metabolized by gut microbiota	May indirectly support barrier via microbial metabolites	Converted to TMA → TMAO in liver → promotes inflammation, steatosis, fibrosis	TMA oxidized by FMO3 → TMAO; gut microbiota-dependent
Carnitine	Meat, dairy; metabolized by gut microbiota	Similar to choline	TMA/TMAO pathway → liver inflammation and fibrosis	Gut bacteria produce TMA → TMAO formation in liver
Lipopolysaccharide (LPS)	Gram-negative bacteria in gut	Leaky gut allows translocation to portal vein	Activates TLR4/CD14 → cytokines ↑ → ROS ↑ → inflammation → fibrosis	Triggers TNF-α, IL-1, IL-6, IL-8, IL-18; Kupffer cell activation
Bile acids (CA, CDCA, DCA, LCA)	Synthesized in liver; modified by gut microbiota	Dysbiosis → barrier weakening	Dysregulated BA metabolism → inflammation, MASLD progression	↑ FFAs → suppress SHP → overactivate CYP7A1 & NTCP → ↑ BA synthesis
Ethanol	Produced by fermentation from gut bacteria (*Escherichia* spp.)	Contributes to barrier disruption	Oxidative stress, inflammation, lipogenesis → hepatic steatosis	↑ Pro-inflammatory cytokines, ROS; alters lipid metabolism

Increase ↑; decreased ↓. SCFAs—Short-chain fatty acids; GLP-1—Glucagon-like peptide-1; PYY—Peptide YY; FIAF—Fasting-induced adipose factor; PPARγ—Peroxisome proliferator-activated receptor gamma; iNOS—Inducible nitric oxide synthase; TMA—Trimethylamine; TMAO—Trimethylamine N-oxide; FMO3—Flavin monooxygenase 3; LPS—Lipopolysaccharide; TLR4—Toll-like receptor 4; CD14—Cluster of differentiation 14; TNF-α—Tumor necrosis factor-alpha; IL—Interleukin; ROS—Reactive oxygen species; CA—Cholic acid; CDCA—Chenodeoxycholic acid; DCA—Deoxycholic acid; LCA—Lithocholic acid; FFAs—Free fatty acids; SHP—Small heterodimer partner; CYP7A1—Cholesterol 7α-hydroxylase; NTCP—Sodium-taurocholate co-transporting polypeptide.

**Table 3:** Key mechanisms linking gut microbiota, microbial metabolites, and MASLD progression.

Mechanism/Factor	Model/Population	Key Changes/Observations	Effect on MASLD	References
Gut microbiota dysbiosis	Humans and animal models	↑ Gram-negative bacteria (*Bacteroidetes*, *Enterobacterales*, *Proteobacteria*); ↓ beneficial bacteria (*Akkermansia muciniphila*, *Faecalibacterium prausnitzii*, *Ruminococcaceae*)	Gut leakiness ↑; LPS translocation ↑; chronic low-grade inflammation ↑; hepatic insulin resistance ↑; de novo lipogenesis ↑; mitochondrial dysfunction ↑; hepatic fat accumulation ↑; MASLD progression ↑	[40,44,48,52]
Intestinal barrier dysfunction	Mice (HFD)	Tight junctions (TJs) ↓; PAMP permeability ↑	Gut leakiness ↑; PAMP translocation ↑; Kupffer cell activation ↑; TLR-mediated signaling ↑; hepatic inflammation ↑; insulin resistance ↑; hepatic steatosis ↑; MASLD progression ↑	[13,26]
High-fat, high-sugar diet	Humans/Animals	Fat accumulation ↑; oxidative stress ↑; intestinal permeability ↑	Intestinal permeability ↑; endotoxemia ↑; oxidative stress ↑; hepatic insulin resistance ↑; de novo lipogenesis ↑; hepatic steatosis ↑; inflammation ↑; MASLD onset and progression ↑	[44,48]
TMAO	Humans	TMAO ↑ via choline/carnitine metabolism	Hepatic fat accumulation ↑; FXR signaling ↓; ER stress ↑; gut barrier dysfunction ↑; bile acid dysregulation ↑; mitochondrial dysfunction ↑; hepatic insulin resistance ↑; pro-fibrotic signaling ↑; MASLD severity ↑	[53,54,55]
SCFAs	Humans/Animals	SCFAs ↓ in dysbiosis; butyrate ↑ colonocyte energy; propionate ↑ gluconeogenesis; acetate ↑ acetyl-CoA	Lipogenesis ↓; fatty acid oxidation ↑; intestinal barrier integrity ↑; inflammation ↓; hepatic steatosis ↓; insulin sensitivity ↑; mitochondrial function ↑; bile acid homeostasis ↑; appetite regulation ↑; MASLD risk ↓	[56,57,58,59]
SCFA epigenetic effects	Humans/Animals	HDAC inhibition ↑; regulatory T cells ↑; TNF-α, IL-6 ↓	Anti-inflammatory effects ↑; hepatic inflammation ↓; fibrosis ↓; immune tolerance ↑; macrophage polarization toward M2 ↑; hepatic stellate cell activation ↓; oxidative stress ↓; hepatocellular injury ↓; MASLD progression ↓	[57,58]
Dysbiosis-related metabolites	Humans	LPS ↑; ethanol ↑	Hepatic inflammation ↑; oxidative stress ↑; endotoxemia ↑; Kupffer cell activation ↑; mitochondrial dysfunction ↑; hepatic insulin resistance ↑; fibrogenic signaling ↑; hepatocyte injury ↑; MASLD progression ↑	[57]

Increased ↑; decreased ↓. MASLD—Metabolic dysfunction-Associated Steatotic Liver Disease; HFD—High-Fat Diet; LPS—Lipopolysaccharide; PAMP—Pathogen-Associated Molecular Pattern; TJs—Tight Junctions; TMAO—Trimethylamine N-oxide; FXR—Farnesoid X Receptor; ER—Endoplasmic Reticulum; SCFAs—Short-Chain Fatty Acids; HDAC—Histone Deacetylase; TNF-α—Tumor Necrosis Factor alpha; IL-6—Interleukin 6.

**Table 4:** Summary of preclinical and clinical studies evaluating the effects of fermented foods on MASLD.

Data	Study Population	Time	Intervention	Key Effects
Kim et al., [60]	Mice models (diet-induced hepatic steatosis), *n* = 20	12 weeks	Fermented dairy products (kefir)	Hepatic lipid accumulation ↓, oxidative stress ↓, inflammatory cytokines ↔
Moreira et al., [61]	Mice with diet-induced obesity and MASLD, *n* not specified	12 weeks	Kombucha	Glucose tolerance ↑, hyperinsulinemia ↓, hepatic steatosis ↓; TNF-α ↓, SREBP-1 ↓; collagen deposition ↓; AKT signaling ↑
Lee et al., [62]	Methionine/choline-deficient MASH mice, *n* = 15	11 weeks	Kombucha	Hepatic triglycerides ↓, inflammation ↓, fibrosis ↓; apoptosis ↓, proliferation ↑; Cd36 ↓, Pparγ ↓, Fas ↓, Srebp1c ↓; β-oxidation genes ↑; FXR/TGR5 signaling modulated
Hyun et al., [63]	Methionine/choline-deficient mice, *n* = 15	4 weeks	Kombucha	Triglyceride ↓, ALT ↓, AST ↓; triglyceride synthesis ↓, fatty acid uptake ↓; liver regeneration ↑
Cardoso et al. [64]	Rats on high-fat, high-fructose diet, *n* not specified	10 weeks	Kombucha with green or black tea	Glucose metabolism ↑, antioxidant capacity ↑; inflammation ↓, NLR ↓, body fat ↓, blood triglycerides ↓; hepatic steatosis ↓; adipogenesis genes modulated, β-oxidation genes ↑
Wastyk et al., [65]	Healthy adults, *n* = 18	10 weeks	Daily fermented foods	Microbiome diversity ↑; inflammatory markers ↓ (including IL-6 and IL-12b)
Han et al., [66]	Women with overweight, *n* = 24	8 weeks	Fermented kimchi	Metabolic markers ↓; gut microbiota composition altered
Yilmaz et al., [67]	IBD patients, *n* = 45	4 weeks	Kefir-based intervention	Inflammatory symptoms ↓
Chen et al., [68]	Women with obesity, *n* = 92	24 weeks	Yogurt	Insulin sensitivity ↑; liver fat accumulation ↓
Nagao et al., [69]	MASLD patients with periodontal disease, *n* = 10	60 days	Amazake	Serum TNF-α ↓; muscle spasms ↓, depression ↓
Mohammadi et al., [70]	Adults with MASLD, *n* = 80	8 weeks	Kefir	HDL cholesterol ↑; systemic inflammation ↓; serum transaminases ↔

Increased ↑; decreased ↓; no change ↔. MASLD—Metabolic Dysfunction-Associated Steatotic Liver Disease; MASH—Metabolic dysfunction-Associated Steatohepatitis; ALT—Alanine Aminotransferase; AST—Aspartate Aminotransferase; TNF-α—Tumor Necrosis Factor alpha; SREBP-1—Sterol Regulatory Element-Binding Protein 1; AKT—Protein kinase B; NLR—Neutrophil-to-Lymphocyte Ratio; FXR—Farnesoid X Receptor; TGR5—Takeda G-protein Receptor 5; Cd36—Cluster of Differentiation 36; Pparγ—Peroxisome Proliferator-Activated Receptor Gamma; Fas—Fatty Acid Synthase; Srebp1c—Sterol Regulatory Element-Binding Protein 1c; Ppargc1α—Peroxisome Proliferator-Activated Receptor Gamma Coactivator 1-alpha; Cpt1—Carnitine Palmitoyltransferase 1; Acox1—Acyl-CoA Oxidase 1; HDL—High-Density Lipoprotein; SCFA—Short-Chain Fatty Acids.

## Data Availability

Data sharing is not applicable to this article as no new data were created or analyzed in this study.

## Abbreviations

The following abbreviations are used in this manuscript:

AGA	American Gastroenterological Association
AKT	Protein Kinase B
ALT	Alanine Aminotransferase
AMPK	AMP-Activated Protein Kinase
AST	Aspartate Aminotransferase
BAs	Bile Acids
BCAAs	Branched-Chain Amino Acids
CA	Cholic Acid
CAP	Controlled Attenuation Parameter
CD14	Cluster of Differentiation 14
CD36	Cluster of Differentiation 36
CDCA	Chenodeoxycholic Acid
CLD	Chronic Liver Disease
CO_2_	Carbon Dioxide
CPT1	Carnitine Palmitoyltransferase 1
CYP7A1	Cholesterol 7α-Hydroxylase
CXCL	C-X-C Motif Chemokine Ligand
DAMPs	Damage-Associated Molecular Patterns
DCA	Deoxycholic Acid
EPS	Exopolysaccharide
ER	Endoplasmic Reticulum
FFAs	Free Fatty Acids
F/B ratio	Firmicutes-to-Bacteroidetes Ratio
FGF15	Fibroblast Growth Factor 15 (mouse)
FGF19	Fibroblast Growth Factor 19 (human)
FIAF	Fasting-Induced Adipose Factor
FIB-4	Fibrosis-4 Index
FMO3	Flavin-Containing Monooxygenase 3
FXR	Farnesoid X Receptor
GLP-1	Glucagon-Like Peptide-1
GPCR	G-Protein Coupled Receptor
GPR41	G-Protein Coupled Receptor 41
GPR43	G-Protein Coupled Receptor 43
GPR109A	G-Protein Coupled Receptor 109A
HCC	Hepatocellular Carcinoma
HDAC	Histone Deacetylase
HDL	High-Density Lipoprotein
HFD	High-Fat Diet
HIF-1α	Hypoxia-Inducible Factor 1 Alpha
IgA	Immunoglobulin A
IL	Interleukin
IL-1β	Interleukin 1 Beta
IL-6	Interleukin 6
IL-8	Interleukin 8
IL-12b	Interleukin 12 Subunit Beta
IL-18	Interleukin 18
iNOS	Inducible Nitric Oxide Synthase
IR	Insulin Resistance
LAB	Lactic Acid Bacteria
LCA	Lithocholic Acid
LPS	Lipopolysaccharide
MASLD	Metabolic Dysfunction-Associated Steatotic Liver Disease
MASH	Metabolic Dysfunction-Associated Steatohepatitis
MRI	Magnetic Resonance Imaging
NAFLD	Non-Alcoholic Fatty Liver Disease
NASH	Non-Alcoholic Steatohepatitis
NLR	Neutrophil-to-Lymphocyte Ratio
NO	Nitric Oxide
NOD2	Nucleotide-Binding Oligomerization Domain 2
NTCP	Sodium Taurocholate Co-Transporting Polypeptide
PAMPs	Pathogen-Associated Molecular Patterns
PPAR	Peroxisome Proliferator-Activated Receptor
PPARγ	Peroxisome Proliferator-Activated Receptor Gamma
PPARgc1α	Peroxisome Proliferator-Activated Receptor Gamma Coactivator 1 Alpha
PYY	Peptide YY
RNA	Ribonucleic Acid
ROS	Reactive Oxygen Species
SCFAs	Short-Chain Fatty Acids
SHP	Small Heterodimer Partner
SREBP-1	Sterol Regulatory Element-Binding Protein 1
SREBP-1c	Sterol Regulatory Element-Binding Protein 1c
TG	Triglycerides
TGR5	Takeda G-Protein Receptor 5
TJs	Tight Junctions
TLR	Toll-Like Receptor
TLR2	Toll-Like Receptor 2
TLR4	Toll-Like Receptor 4
TMA	Trimethylamine
TMAO	Trimethylamine N-Oxide
TNF-α	Tumor Necrosis Factor Alpha
VLDL	Very-Low-Density Lipoprotein
YY1	Yin Yang 1
ZO-1	Zonula Occludens-1
